# Toll-Like Receptor 3 Signalling Up-Regulates Expression of the HIV Co-Receptor G-Protein Coupled Receptor 15 on Human CD4^+^ T Cells

**DOI:** 10.1371/journal.pone.0088195

**Published:** 2014-02-18

**Authors:** Miriam Kiene, Bence Rethi, Marianne Jansson, Stephanie Dillon, Eric Lee, Rebecka Lantto, Cara Wilson, Stefan Pöhlmann, Francesca Chiodi

**Affiliations:** 1 Institute of Virology, Hannover Medical School, Hannover, Germany; 2 Department of Microbiology, Tumor and Cell Biology, Karolinska Institutet, Stockholm, Sweden; 3 Infection Biology Unit, German Primate Center, Göttingen, Germany; 4 Department of Laboratory Medicine, Lund University, Lund, Sweden; 5 Division of Infectious Diseases, University of Colorado Anschutz Medical Campus, Aurora, Colorado, United States of America; Emory University School of Medicine, United States of America

## Abstract

**Background:**

Many HIV-2 and SIV isolates, as well as some HIV-1 strains, can use the orphan 7-transmembrane receptor GPR15 as co-receptor for efficient entry into host cells. GPR15 is expressed on central memory and effector memory CD4^+^ T cells in healthy individuals and a subset of these cells is susceptible to HIV-1 and SIV infection. However, it has not been determined whether GPR15 expression is altered in the context of HIV-1 infection.

**Results:**

Here, we show that GPR15 expression in CD4^+^ T cells is markedly up-regulated in some HIV-1 infected individuals compared to the rest of the infected patients and to healthy controls. Infection of the PM1 T cell line with primary HIV-1 isolates was found to up-regulate GPR15 expression on the infected cells, indicating that viral components can induce GPR15 expression. Up-regulation of GPR15 expression on CD4^+^ T cells was induced by activation of Toll-like receptor 3 signalling via TIR-domain-containing adapter-inducing interferon-β (TRIF) and was more prominent on gut-homing compared to lymph node-homing CD4^+^ T cells.

**Conclusion:**

These results suggest that infection-induced up-regulation of GPR15 expression could increase susceptibility of CD4^+^ T cells to HIV infection and target cell availability in the gut in some infected individuals.

## Introduction

The envelope glycoprotein (Env) of the simian and human immunodeficiency virus (HIV and SIV) mediates host cell entry. For this purpose, Env interacts with CD4 and a co-receptor, usually CCR5 and/or CXCR4 [1]. Sexually transmitted HIV-1 is usually restricted to CCR5 (R5), although rare transmissions of CXCR4-using (monotropic X4 or dualtropic R5X4) viruses have been reported [2], [3]. During the later stages of the infection, viruses with the ability to use CXCR4 emerge in a large proportion of infected patients, depending on HIV-1 subtype, and the emergence of these viruses has been associated with a poor clinical prognosis [4], [5]. In addition to the major co-receptors, CCR5 and CXCR4, a variety of structurally related 7-transmembrane receptors, collectively termed as alternative co-receptors, are frequently used by SIV and HIV-2 for efficient entry into cell lines [6]–[10]. Some HIV-1 isolates can also engage alternative co-receptors for cellular entry but usage of these receptors is less frequent compared to HIV-2 and SIV [8], [10]–[14]. There is currently little evidence that alternative co-receptors can support HIV-1 spread *in vivo*, although some studies found that alternative co-receptors can promote infection of peripheral blood mononuclear cells by certain HIV-1 isolates [15], [16]. However, it is conceivable that targeting of CCR5 and CXCR4 [17] during HIV-1 antiretroviral therapy could potentially select for viruses which either use CCR5/CXCR4 in the drug-bound state or engage alternative co-receptors for entry.

The orphan 7-transmembrane receptor GPR15 is used by many HIV-2 and SIV strains, as well as some HIV-1 isolates for efficient infection of co-receptor transfected cell lines [6], [9], [13], [18]–[20]. However, it is not completely understood whether GPR15 is expressed on viral target cells and contributes to viral spread in the infected host. A V3-loop mutation which impairs the ability of SIV_mac239_ to use GPR15 but not to use CCR5 for cell entry does not impair SIV replication and pathogenesis in infected rhesus macaques [21]. This suggests that GPR15 is dispensable for viral spread provided that CCR5 usage is preserved. However, a transmitted founder HIV-1 variant was described which was impaired in its ability to use CCR5 and CXCR4 for viral entry but could instead use GPR15 *in vitro* [22]. GPR15 is expressed on CD4^+^ T cells, especially on cells with central memory and effector memory phenotype [23] and the molecule has recently been shown to be important for T cell homing to the colon laminar propria in mice [24]. Expression of CXCR4 can be regulated by Toll-like receptor 3 (TLR3) signalling [25], suggesting that co-receptor expression might be modulated upon recognition of HIV and SIV by components of the innate immune system. Phosphoinositide-3 kinase (PI3K) activation induces GPR15 surface expression [26] and PI3K can be activated via the TLR3 signalling pathway [27], suggesting that GPR15 expression might be regulated by TLR3 signalling. However, the impact of TLR activation on GPR15 expression has not been determined and it is unknown if GPR15 expression is altered in the context of HIV-1 infection.

Here, we show that TLR3 stimulation of peripheral blood T cells increases GPR15 surface expression and that the co-receptor is highly up-regulated on central memory and effector memory CD4^+^ T cells in some HIV-1 infected patients compared to non-infected controls. Infection of the PM1 T cell line with different HIV-1 primary isolates showed that GPR15 expression can be up-regulated in productively infected cells. In addition, we report that TLR3-induced GPR15 up-regulation is more prominent in gut homing CD4^+^ peripheral T cells than in the T cells homing to lymph nodes, suggesting that TLR3-dependent up-regulation of GPR15 could increase HIV infection of intestinal T cells. Indeed the intestinal CD4^+^ T cells show high expression of GPR15 particularly those in the lamina propria. This suggests that infection induced TLR3 stimulation could increase GPR15 homing of T cells to the gut thereby increasing the target cell availability and facilitating viral dissemination.

## Materials and Methods

### Study Participants

Blood samples were obtained from healthy donors and HIV-1 infected patients. The age of the non-infected controls (n = 25) ranged from 29 to 49 years (50% males). The age of the HIV-1 infected individuals (n = 11; all males) ranged from 31 to 57 years. The CD4^+^ T cell count was between 62 to 903 cells/µl and the viral load in the three patients naïve to treatment was 1230, 11300 and 100000 HIV RNA copies/ml. The blood specimens were collected from HIV-1 infected individuals at the South Hospital, Stockholm; the study participants provided their written informed consent to participate in this study. The ethical committee at Karolinska Institutet approved the study involving human blood and patient material.

Colon biopsies were obtained by flexible sigmoidoscopy from 3 HIV-1-infected and 3 healthy subjects who were enrolled in a larger clinical study at the University of Colorado Anschutz Medical Campus. All HIV-infected subjects were receiving care at the University of Colorado Infectious Disease Group Practice, University of Colorado Hospital and were anti-retroviral treatment (ART) naïve. Their CD4 T cell counts were 363, 400 and 532 cells/µl and their plasma viral loads 8400, 26000 and 207000 RNA copies/ml. All subjects voluntarily gave written, informed consent. The clinical study was approved by the Colorado Multiple Institutional Review Board (COMIRB) at the Anschutz Medical Campus.

### Cell culture

Peripheral blood mononuclear cells (PBMCs) were isolated by Lymphoprep (Axis shield PoC As, Oslo, Norway) gradient centrifugation and were cultured in RPMI 1640 containing 10% FCS and antibiotics penicillin/streptomycin (all HyClone Thermo scientific, Logan, USA). The PM1 T cell line (NIH AIDS repository) was cultured similarly but with medium supplemented with 2.5% FCS.

T and B cells were separated using Pan T cell separation Kit and B cell separation Kit II (Milteny Biotec, Bergisch Gladbach, Germany) following the manufacturer's instructions.

### TLR stimulation

PBMCs were seeded at 1×10^6^ cells/ml in a 24-well plate and stimulated with the following TLR ligands at the concentration of 1 µg/ml Pam3CSK4 (TLR2), 10 µg/ml polyIC (TLR3), 0.5 µg/ml LPS-EB ultrapure (TLR4), 1 µg/ml CLO75 (TLR7/8), 4 µg/ml CpG (ODN 2006) (TLR9) (all Invivogen, San Diego, CA, USA) or PBS (HyClone, Thermo Scientific) for 24 hours.

For inhibition of TLR3 signalling, PBMCs were pre-treated for 6 hours with 5 µM PepinhTRIF (Invivogen) and subsequently stimulated with polyIC as described above.

### Virus infection assay

PM1 cells [28] were infected with three different primary HIV-1 isolates 25, 2195 and 4052 [11], [62] for three days. 2.5×10^4^ cells were infected in triplicates in a 96- well format. After 24 hours cells were washed once with PBS and supplemented with fresh medium. On day 3 triplicates were pooled and stained for GPR15 and intracellular p24 as indicated below.

### FACS staining

For analysis of GPR15 or CXCR6 expression, a total number of 5×10^5^ PBMCs or PM1 cells were washed with FACS buffer [1× PBS supplemented with 5% FCS (HyClone Thermo scientific)], blocked for 30 min with 50 µl human serum and stained with mouse anti-GPR15/CXCR6 antibody or matched mIgG2b isotype control antibody (both R&D Systems, Minneapolis, MN, USA) diluted 1∶100 in FACS buffer for 30 min at 4°C. The cells were washed again and stained with anti-mouse Dylight649 (1∶250) or Dylight488 (1∶200) (Dianova, Hamburg, Germany) secondary antibody in FACS buffer for 30 min at 4°C, followed by washing and fixation in PBS/1% paraformaldehyde. For staining of CXCR4 and CCR5, 1.25 µl of anti-CXCR4 (BD) and 10 µl of anti-CCR5 antibodies (R&D System) together with matched isotype controls were used.

For analysis of GPR15 expression on CD4^+^ T cell subpopulations GPR15 staining was performed as described above using anti-mouse Dylight649 secondary antibody followed by a third staining step with 2.5 µl anti-CD3 V450, 5 µl anti-CD4 PE, 5 µl anti-CD45RA PerCP-Cy5.5 and 1 µl anti-CCR7 PE-Cy7 (all BD, Franklin Lakes, NJ, USA).

For GPR15 staining after TLR stimulation Dylight488 was used as secondary antibody. For analysis of GPR15 surface expression on different lymphocyte subsets PBMCs were stained with 1 µl anti-CD4 PE-Texas Red (Abcam, Cambridge, UK), 2.5 µl anti-CD8 APC and 5 µl anti-CD19 PE antibodies (all BD).

To analyse the co-expression of GPR15 with lymph node and gut homing receptors, PBMCs were stained directly *ex vivo* using 5 µl anti-CD62L PE, 10 µl anti-α4 integrin PE (CD49d) and 10 µl anti-β7 integrin APC antibodies (all BD), and 1 µl CD4 PE-Texas Red (Abcam).

For analysis of GPR15 expression on HIV-1 infected PM1 cells the cells were stained for GPR15 as indicated above. Afterwards the cells were permeabilised and stained for intracellular p24 using the cytofix/cytoperm kit (BD Biosciences) following the manufactures instructions. The intracellular p24 staining was performed for 20 min at 4°C with 5 µl KC57-RD1 (Beckman Coulter) 1∶10 diluted in BD permWash. After washing cells were fixed in 4% PFA.

All stainings were analyzed via LSRII (BD Biosciences, San José, CA, USA) and FlowJo (Tree Star Inc., Ashland, OR, USA) software.

### Evaluation of GPR15 expression in colon tissue

#### FACS staining

Human colon tissue samples were obtained from patients (n = 2) undergoing elective abdominal surgery, representing otherwise discarded tissue and considered macroscopically normal as previously described [63], [64]. All patients undergoing surgery signed a release to allow the unrestricted use of discarded tissues for research purposes, and all protected patient information was de-identified to the laboratory investigators. This research was reviewed by COMIRB at the University of Colorado Anschutz Medical Campus and was granted exempt research status.

Intraepithelial mononuclear cells (IEMC) and lamina propria mononuclear cells (LPMC) were isolated from tissue samples and released IEMC or LPMC were cryopreserved and stored in liquid nitrogen as detailed elsewhere [63], [64]. For analysis of GPR15 expression, cells were first stained with mouse anti-GPR15/CXCR6 antibody or matched mIgG2b isotype control antibody (both R&D Systems, Minneapolis, MN, USA) as detailed above, followed by staining with PerCp-Cy5.5 CD45 (eBioscience, San Diego, CA), ECD CD3 (Beckman Coulter, Fullerton, CA), eFluor^450^ CD4 (eBioscience), AF647 anti-mouse IgG and a Live/Dead Fixable Dead Cell Stain (Aqua, Invitrogen) as previously described [65].

#### Immunohistology staining

Colon biopsies were snap frozen in OCT (optimal cutting temperature, Tissue Tek) and 7 um thick sections were cut and mounted onto slides. Samples were fixed with 1% paraformaldehyde (PFA) and stained overnight at 4°C with anti-GPR15 (Abcam 1∶100) followed by labeling with anti-rabbit Alexa488 (Molecular Probes, 1∶400 dilution). Samples were additionally stained for 1 h at room temperature with anti-CD4 (BD Biosciences, 1∶200 dilution) followed by labeling with anti-mouse Alexa647 (Molecular Probes, 1∶400 dilution). Lastly, samples were mounted and preserved with Prolong Gold containing DAPI, which stains cell nuclei (Invitrogen). Images were acquired on a Zeiss LSM510 META confocal using sections stained only with secondary labels to set the background threshold. Three biopsies per patient and 15–20 images per biopsy were acquired at 63×. Images were analyzed and enumerated using ImageJ (National Institute of Health) cell counting software in a blinded fashion.

### Statistical Methods

All error bars represent standard deviations. Significances were calculated using student's t-Test or Wilcoxon signed-rank test with Prism (version 5.0b; GraphPad, La Jolla, CA, USA).

## Results

### GPR15 expression on T cells is up-regulated in some HIV-1 infected individuals

**Figure 1:**
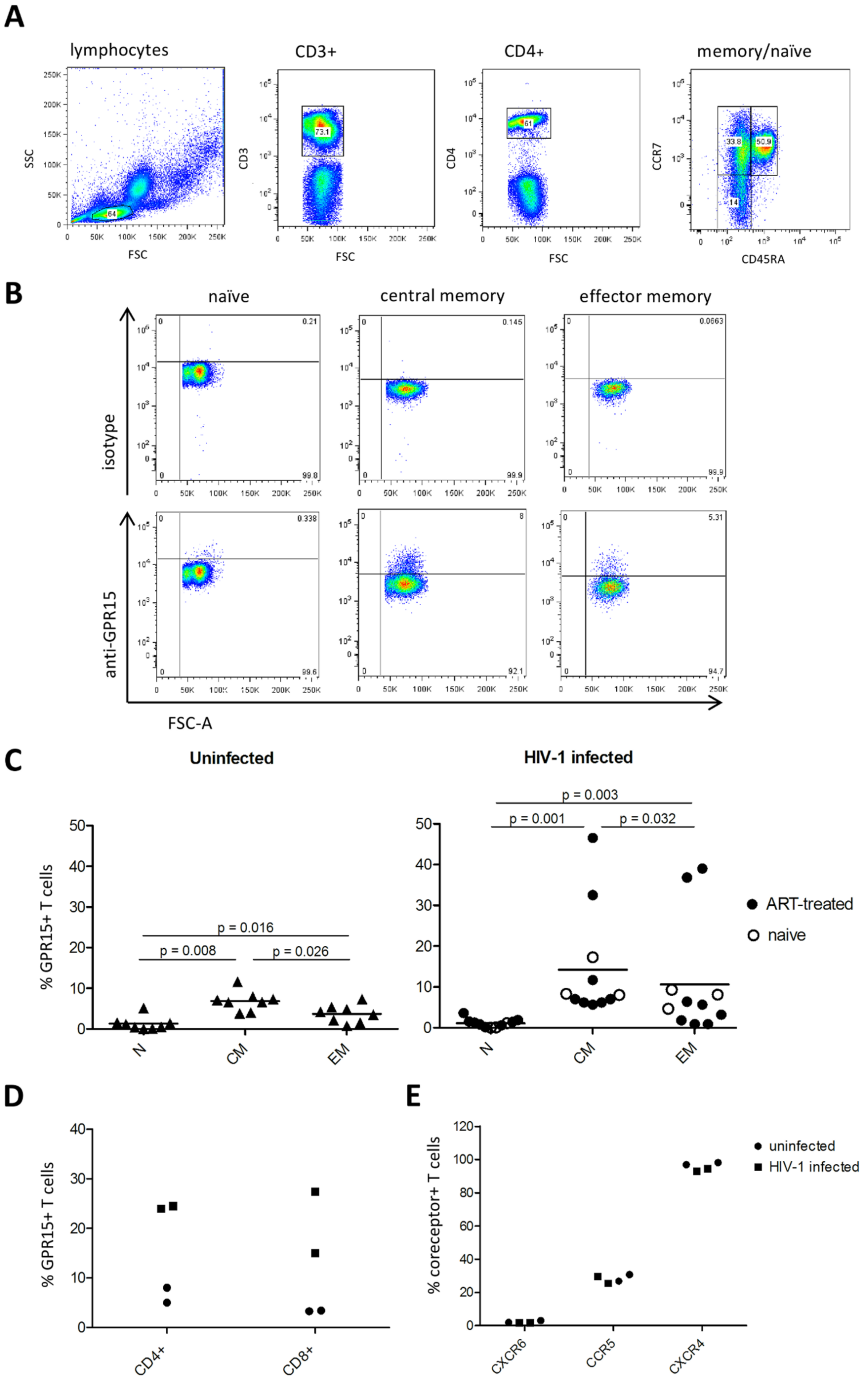
GPR15 is mostly present on central memory CD4^+^Tcells in HIV-1 infected individuals and uninfected controls. PBMCs were isolated from whole blood by Lymphoprep gradient centrifugation and stained for CD3, CD4, CCR7, CD45RA and GPR15. (A) Cells were gated for lymphocytes, CD3^+^, CD4^+^ and CCR7^+^CD45RA^−^ (CM: central memory), CCR7^−^CD45RA^−^ (EM: effector memory) or CCR7^+^CD45RA^+^ (N: naïve) (A) and GPR15 expression on the subsets was analyzed via FACS (B). The GPR15 expression on CD4^+^ T cell subpopulations was analyzed in eight uninfected controls and eleven HIV-1 infected patients (C) as indicated in (A, B). GPR15 expression is shown as the percent of the analysed subpopulation which expresses the co-receptor (C). Blood samples taken two month later from the two high GPR15 expressing HIV-1 infected patients and two controls (shown in C) were stained for GPR15, CD4 and CD8 (D) or CD4 and other co-receptors like CXCR4, CCR5 and CXCR6 (E). The co-receptor expressions are shown as a percent of CD4^+^ and CD8^+^ T cells expressing GPR15 (D) or of CD4^+^ T cells expressing CXCR4, CCR5 or CXCR6 (E). Statistical analysis was done using Wilcoxon signed-rank test with GraphPad Prism.

We have previously reported that GPR15 is expressed on central memory and, to a lesser extent, on effector memory T cells while expression of this receptor on naïve T cells is absent [23]. To investigate whether GPR15 expression on these T cell subsets is altered in the context of HIV-1 infection, we analyzed surface expression of GPR15 on CCR7^+^CD45RA^−^ central memory, CCR7^−^CD45RA^−^ effector memory and CCR7^+^CD45RA^+^ naïve CD4^+^ T cells (Figures 1A and 1B) from healthy donors and HIV-1 infected individuals. In line with our previous results [23], GPR15 was expressed to a significantly higher level on central memory (7±2.4%) compared to effector memory (3.7±2.2%; p = 0.026) and naïve CD4^+^ T cells (no detectable expression) (p = 0.016) in non-infected controls. A similar expression pattern was observed for HIV-1 infected individuals (ART treated and non-treated individuals) (Figure 1C), with no significant differences when comparing the levels to the non-infected controls. Although a limited number of specimens obtained from ART naïve patients was used in the study, we could not find a significant difference in GPR15 expression whencomparing ART treated HIV-1 infected individuals to the non-treated ones. In the HIV-1 group two patients exhibited markedly increased GPR15 expression, with up to 46.5% of cells in the central memory subset and up to 39% of cells in the effector memory subset expressing GPR15 (Figure 1C). To exclude that environmental factors at the time point of blood drawing influenced GPR15 expression, samples from the two patients with high GPR15 expression were collected 2 month later and again analyzed for GPR15 surface levels. GPR15 expression on CD4^+^ from the two patient specimens was still markedly higher than GPR15 levels on cells from two uninfected controls (Figure 1D) and similar results were obtained for CD8^+^ T cells (Figure 1D). The viral load and age was not different between HIV-1 infected individuals expressing high and average levels of GPR15 and the reason for the increased GPR15 expression in some patients is at present unclear. Finally, we assessed whether the expression of other co-receptors, CXCR6, CCR5 and CXCR4, also was modulated in the context of HIV-1 infection. The expression of these receptors was not altered in the HIV-1 infected compared to the uninfected controls (Figure 1E). Thus, the highest expression of GPR15 in healthy individuals and HIV-1 patients is found on central memory T cells and some HIV-1 patients' exhibit markedly elevated GPR15 levels.

### Infection with primary HIV-1 isolates induces GPR15 expression on the infected PM1 T cells

**Figure 2:**
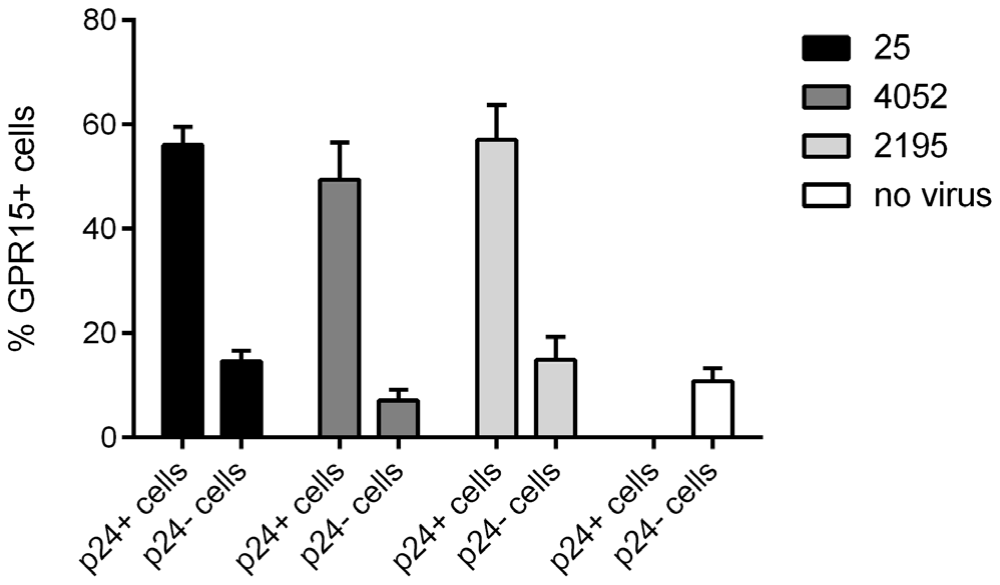
HIV-1 infection increases GPR15 expression on infected cells. The PM1 T cell line was infected with three different primary HIV-1 isolates the multitropic isolates 25 and 4052 and the R5-tropic 2195 for 3 days and afterwards stained for GPR15 surface expression and intracellular p24. The percent of uninfected (p24−) or infected (p24+)cells expressing GPR15 is shown.

To investigate if HIV-1 infection can up-regulate GPR15 surface expression we chose the PM1 T cell line as a model system [28].. No appreciable change in total GPR15 expression was observed on non-infected cells upon infection with the multitropic HIV-1 isolates 25, 4054 and the R5 tropic isolate 2195. All three isolates infected PM1 cells with comparable efficiency. For all three virus isolates the expression of GPR15 was markedly higher on infected compared to uninfected PM1 cells, as determined by co-staining of intracellular HIV-1 p24 (Figure 2), suggesting that HIV-1 infection up-regulates GPR15 expression.

### TLR3 stimulation up-regulates GPR15 expression on T cells

**Figure 3:**
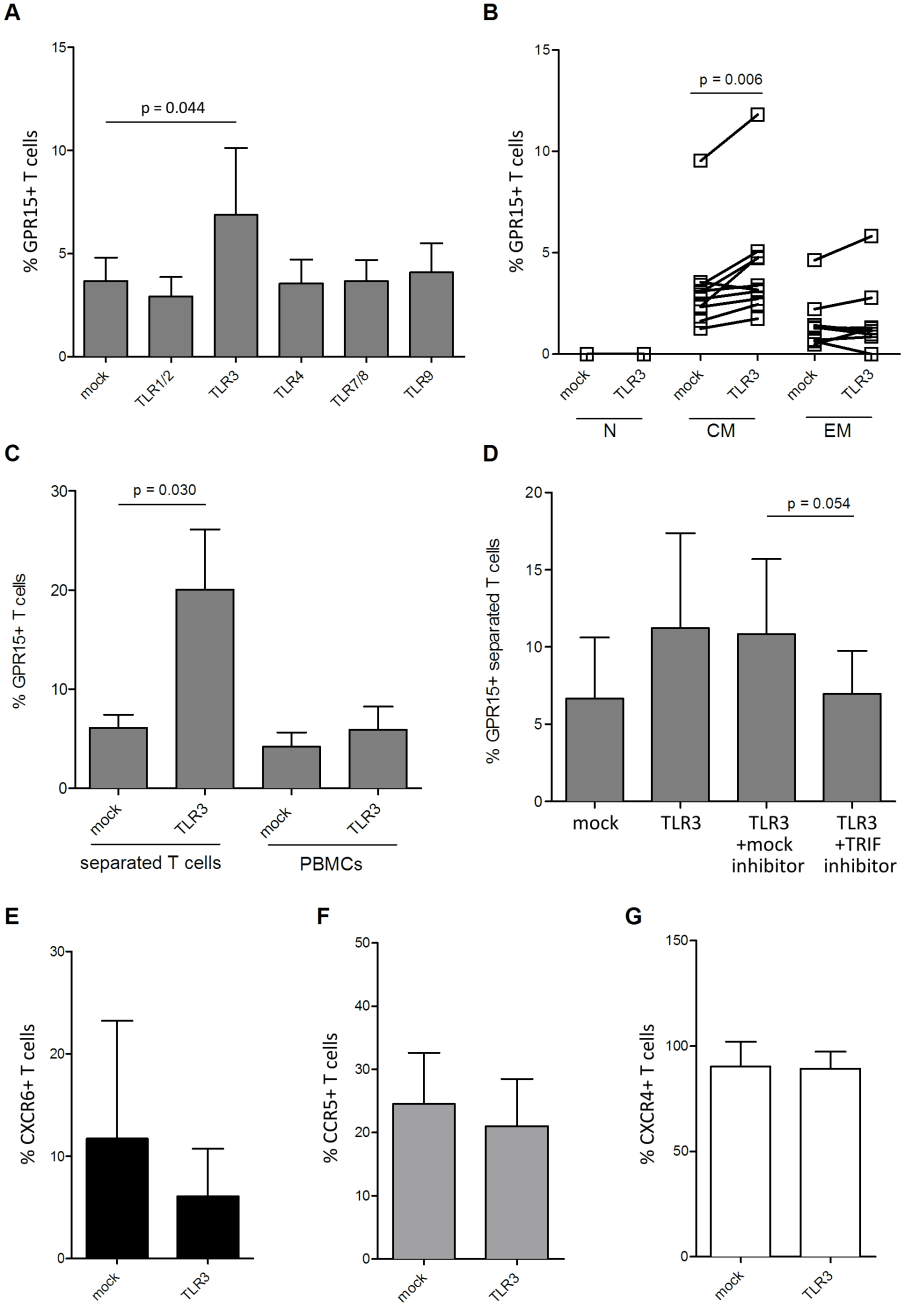
TLR3 stimulation increases GPR15 on the surface of CD4^+^ T cells. PBMCs were incubated with different ligands for TLRs and GPR15 expression on CD4^+^ T cells was analyzed. Cells were gated for lymphocytes, CD3^+^, CD4^+^ as already shown in Figure 1A. The bar graph is a summary of four donors which were analysed in two independent experiments (A). Upon TLR3 triggering, GPR15 is mostly up-regulated on central memory T cells (B). To exclude cell-cell interaction effects T cells were further separated using negative selection with magnetic beads and stimulated with TLR3 ligand polyIC (C). Pre-treatment of T cells with TLR3 signalling inhibitor PepinhTRIF abrogates the increase of GPR15 on the T cell surface (D). To test if TLR3 stimulation can up-regulate other co-receptors it was also stained for CXCR6 (E), CCR5 (F) and CXCR4 (G). GPR15 expression is shown as a percent of the gated CD4^+^ T cell subpopulations. Statistical analysis was done with GraphPad Prism using paired t-test.

In order to explore potential mechanisms underlying the up-regulation of GPR15 expression in some HIV-1 patients and by HIV-1 primary isolates in cell culture, we next assessed whether TLR ligands alter GPR15 surface expression on lymphocytes. Human T cells express different TLRs including TLR 1, 2, 3, 4, 7, 8 and 9 [29]–[31] of which TLR 3, 7, 8 and 9 recognize viral components [32]. We assessed GPR15 expression upon TLR stimulation with the TLR1/2 ligand Pam3CSK4 (synthetic triacylated Lipopeptide), the TLR3 ligand polyIC (synthetic analogue of dsRNA), the TLR4 ligand bacterial LPS, the TLR7/8 ligand CLO75 (synthetic analogue of ssRNA) and the TLR9 ligand CpG (synthetic oligodeoxynucleotide). Treatment of cells with the TLR3 ligand polyIC up-regulated GPR15 expression while expression was not modulated by the other TLR agonists tested (Figure 3A). The GPR15 up-regulation was most prominent on central memory CD4^+^ T cells (p = 0.006), while the effect was less prominent on effector memory and absent on naïve T cells (Figure 3B). To exclude the possibility that the interaction of T cells with other cells within the PBMC analyzed was responsible for the increase in GPR15 expression on CD4^+^ T cells, we isolated T cells prior to TLR3 stimulation (Figure 3C). Stimulation of TLR3 on isolated CD4^+^ T cells led to a 3-fold increase (6.1±1.3 to 20.1±6.1%) of GPR15 expression (p = 0.03). Thus, TLR3 stimulation directly up-regulates GPR15 expression on CD4^+^ T cells.

The TLR3 signaling pathway requires the down-stream activity of the TRIF adaptorprotein [32]. We thus tested if inhibition of TRIF abrogates the increase of GPR15 expression upon TLR3 stimulation. CD4^+^ T cells treated with the TRIF inhibitor PepinhTRIF did not up-regulate GPR15 upon TLR3 stimulation, as compared to the cells treated with the control peptide (p = 0.054) (Figure 3D). Thus, up-regulation of GPR15 expression upon TLR3 ligation is TRIF-dependent. To exclude that TLR3 stimulation unspecifically up-regulates co-receptor expression, we tested if TLR3 stimulation up-regulates expression of CXCR4, CXCR6 and CCR5. However, none of the co-receptors tested showed an increase in expression after polyIC treatment (Figure 3E–G).

### TLR3 induces GPR15 up-regulation on CD8^+^ T cells and CD19^+^ B cells

**Figure 4:**
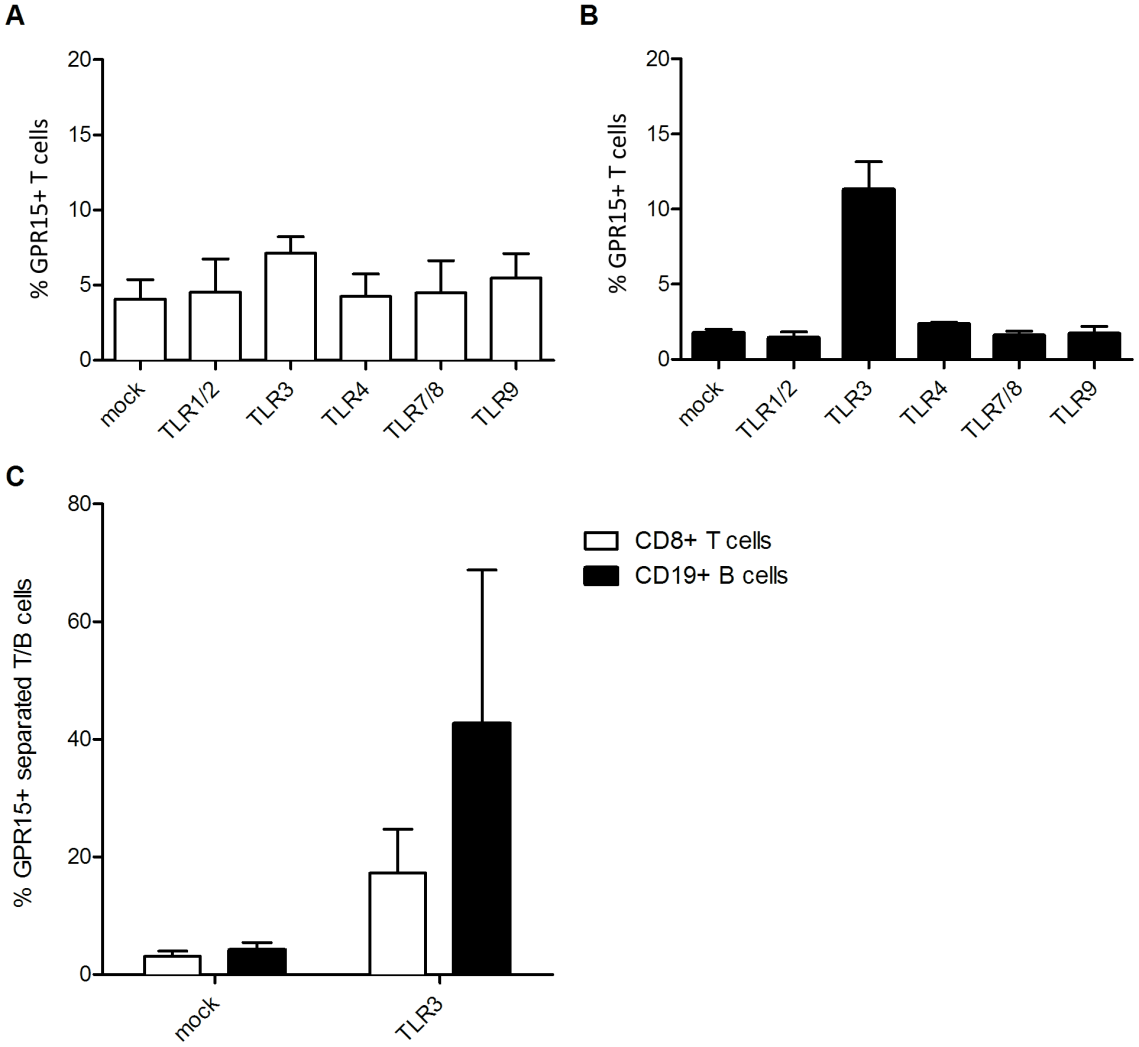
TLR3 stimulation up-regulates GPR15 also on CD8^+^ T cells and CD19^+^ B cells. The GPR15 expression was studied on whole PBMCs (A,B) or separated T and B cells (C) using additional anti-CD8 (A) or CD19 (B) antibodies. GPR15 is shown as a percent of CD8^+^ T or CD19^+^ B cells.

We next asked if up-regulation of GPR15 expression upon TLR3 ligation is specific for CD4^+^ T lymphocytes or can also be detected on CD8^+^ and CD19^+^ lymphocytes within total PBMCs (Figure 4A,B). Stimulation of PBMCs with polyIC up-regulated GPR15 expression on CD8^+^ T cells (4.1±1.3 to 7.1±1.1%) (Figure 3A) and particularly on CD19^+^ B cells (1.75±0.25 to 11.3±1.8%) (Figure 4B). Similar results were obtained with isolated CD8^+^ T cells (3.1±0.9 to 17.3±7.5%) and CD19^+^ B cells (4.2±1.3 to 42.7±26%) (Figure 4C), indicating that the GPR15 up-regulation due to TLR3 engagement up-regulates GPR15 expression on diverse sub-populations of lymphocytes. TLR3 signaling leads to interferon-β (IFN-β) expression [32]. To prove that the downstream IFN-β expression is not responsible for the GPR15 up-regulation we cultured PBMCs in the presence of 20 ng/ml IFN-β for 24 hours. GPR15 expression of peripheral T and B lymphocytes was not affected by the presence of recombinant IFN-β (relative mean fold-change in the frequency of GPR15^+^ T and B lymphocytes were 1.06±0.6 and 1.05±0.8 respectively) (data not shown). These results indicated that the stimulatory effect of polyIC on GPR15 expression is type-I IFN independent.

### GPR15 up-regulation is selective for gut-homing CD4^+^ T cells and the co-receptor is highly expressed in colon CD4^+^ T cells

**Figure 5:**
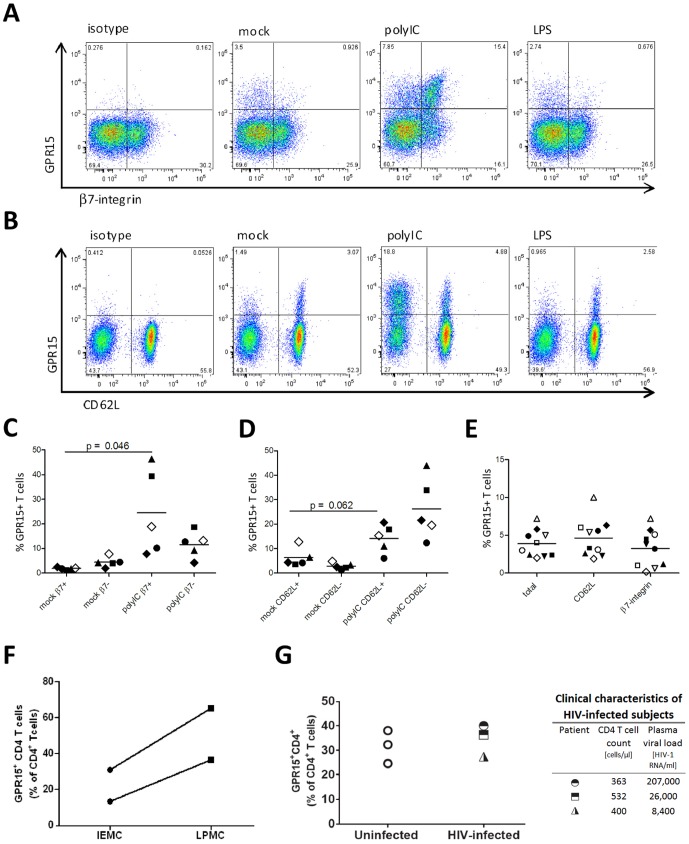
GPR15 is strongly up-regulated on gut homing CD4^+^Tcells and is highly expressed on colon CD4^+^Tcells. TLR3 stimulation up-regulates GPR15 on gut homing (α4β7-integrin^+^) (A, C) and on CD4^+^ T cells homing to lymph nodes (CD62L^+^) (B, D). The different symbols in the Figures C and D specify different donors. Before TLR3 stimulation both subsets express GPR15 to a similar level (E). The different symbols describe individual donors (E). PBMCs were isolated from whole blood by Lymphoprep gradient centrifugation and co-stained for CD4, GPR15 and CD62L or β7-integrin. The cells were gated on lymphocytes, CD4^+^ as shown in Figure 1 A. The graphs show GPR15 expression as a percent of CD62L^+^ CD4^+^ T cells or β7^+^ CD4^+^ T cells expressing the co-receptor. The experiments were done at least two times including two donors each time. Statistical analysis was done as described in Figure 3 using paired t-test. Human colon intraepithelial mononuclear cells (IEMC) and lamina propria mononuclear cells (LPMC) express GPR15 on high level. IEMC and LPMC were isolated following the described protocol and co-stained for CD45, CD3, CD4 and GPR15. Cells were gated as described in Figure 1A with the accretion that CD45 were gated out to exclude epithelial cell contamination (F). Colon biopsies of HIV-1 infected and uninfected individuals were immunofluorescently stained for GPR15, CD4 and cell nuclei using DAPI. Slides were analysed by confocal microscopy. Three biopsies per patient and 15–20 images per biopsy were acquired at 63×. Cells were enumerated using ImageJ cell counting software for % of CD4^+^ cell expressing GPR15 (G).

The gut associated lymphoid tissue (GALT) is the major site of HIV-1 replication and CD4^+^ T cell depletion [33]–[38]. Since the gut mucosal homing receptor α4β7-integrin binds to HIV-1 and might be important for productive infection of CD4^+^ T cells [39], we next investigated whether GPR15 positive lymphocytes co-express gut (α4β7-integrin) and lymph node (CD62L) homing receptors. To verify that all β7-integrin positive cells are also positive for α4-integrin a double staining was performed as a control (not shown). Prior to stimulation, a similar percentage of CD4^+^ T expressing gut or lymph node homing receptors co-expressed GPR15 (lymph node homing: 4.7±2.5%, gut-homing: 3.3±2.4%) (Figure 5E). GPR15 expression on gut homing T cells was significantly up-regulated upon polyIC treatment (Figures 5A and 5C). In contrast, polyIC treatment had only a minor effect on GPR15 expression on lymph node homing cells, which did not reach statistical significance (p = 0.062) (Figure 5D). Thus, among CD4-positive T cells, the TLR3 induced up-regulation of GPR15 expression is largely specific for gut homing CD4^+^ T cells and might broaden the target cell availability during HIV-1 infection in the gut.

Since HIV-1 highly replicates in the GALT and we found a significant GPR15 up-regulation on gut homing CD4^+^ T cells upon TLR3 stimulation, we assessed the GPR15 expression on normal intestinal CD4^+^ T cells (n = 2). Colon tissue lamina propria and intraepithelial T cells were separated from surgical colon samples and stained for CD45, CD3, CD4 and GPR15. The intestinal CD4^+^ T cells express high levels of GPR15 with expression levels being particularly high in lamina propria derived CD4^+^ T cells when compared to intraepithelial derived colon CD4^+^ T cells (Figure 5F). To investigate if GPR15 is up-regulated in the gut tissue of HIV-1 infected individuals colon biopsies from 3 uninfected patients and 3 untreated, viremic HIV-1 infected patients were co-stained for CD4 (red) and GPR15 (green) (Figure 5G). No significant difference in percentage of GPR15-expressing CD4^+^ T cells could be detected between the uninfected and the HIV-1 infected samples (Figure 5H), indicating that GPR15 expression on intestinal CD4^+^ T cells is robust and not additionally augmented upon HIV-1 infection.

## Discussion

In this study we investigated whether the expression of HIV co-receptor GPR15 is altered in immune cells of HIV-1 infected patients. We focused on CD4^+^ T cells since these are the main target cells for HIV infection [40]. We found an up-regulation of GPR15 expression in some HIV-1 infected patients. In addition we observed that upon *in vitro* HIV-1 infection, PM1 T cells showed increased GPR15 levels on the productively infected cells suggesting a role of viral components in up-regulating GPR15 expression. Further experiments showed that activation of Toll-like receptor 3 signalling via TRIF adaptor proteins was inducing GPR15 expression in CD4^+^ T cells. The co-receptor up-regulation was more prominent on gut homing compared to lymph node homing CD4^+^ T cells and GPR15 was highly expressed in colon CD4^+^ T cells. These results suggest that HIV-1 infection likely via TLR3 stimulation up-regulates GPR15 inducing gut homing of T cells. This in turn could increase target cell availability in the gut and support viral dissemination in some HIV infected individuals.

We previously showed that GPR15 is mostly expressed on central memory T cells in the blood [23], a finding that was corroborated in the present study. Since the main co-receptors for viral entry CCR5 and CXCR4 are mostly found on effector memory or naïve CD4^+^ T cells [41], [42], the expression of GPR15 on central memory T cells potentially expands the target cell population of HIV/SIV to bigger part of another CD4^+^ T cell subpopulation. Central memory CD4^+^ T cells are a particularly interesting target cell population since transmitted viral variants do mostly not use CXCR4 [2], which is present on naïve T cells travelling to the lymph nodes; effector memory cells expressing CCR5 on the other hand remain at their effector sites since they lose expression of the lymph node homing chemokine receptor CCR7 [41], [43], [44]. Indeed it has recently been shown that central memory like CD4^+^ T cells are important for dissemination of the virus from mucosal surfaces, the site of viral entry into the patient, into the lymphoid tissue, the major site of viral replication [45]. Thus, according to this scenario and to our results showing that GPR15 is mostly expressed in central memory CD4^+^ T cells, entry into central memory T cells mediated by GPR15 could contribute to viral dissemination. However, the contribution of GPR15 entry to virus spread might be more prominent in HIV-2 infected patients and in the SIV model system since GPR15 usage is more frequent among HIV-2 and SIV isolates compared to HIV-1 isolates [6]–[10].

We found that GPR15 expression was up-regulated *ex vivo* on CD4^+^ T cells in two out of eleven HIV-1 patients, that HIV-1 infection can upregulate GPR15 in PM1 T cells and that TLR3 triggering by dsRNA [46] up-regulated GPR15 expression on CD4^+^ T cells. Despite the fact that HIV-1 is a single stranded RNA virus, HIV-1 can potentially trigger TLR3 activation since it produces dsRNA intermediates during its replication cycle [47] and since a synthetic analogue of ssRNA was found to induce TLR3 signalling [48]. Moreover, previous reports showed up-regulation of TLR3 expression in lymph nodes during SIV infection [49] and in PBMCs of HIV infected patients with advanced disease [50]. This in turn could explain a higher sensitivity in someHIV infected patients to TLR3 triggering. Ongoing virus replication despite ART has been proposed to take place during HIV-1 infection [51] supporting the possibility that TLR3 stimulation by HIV-1 could take place during ART treatment. The up-regulation of GPR15 could support infection, more likely super-infection as well as viral dissemination. Whether increased co-receptor expression results in augmented susceptibility to HIV-1 infection remains to be established.

TLR3 is likely located in the endosome [52], [53], which is communicating with the extracellular matrix. Thus, it is conceivable that TLR3 senses extracellular viral RNA, for example RNA released from dying infected cells. Indeed, massive memory CD4^+^ T cell death due to direct infection and bystander cell apoptosis has been reported [54]. Phagocytosis has so far only been shown for γδ T cells [55] however indirect evidence that TLR3 can be generally stimulated in CD4^+^ T cells is given by a study showing that stimulation with polyIC can up-regulate costimulatory molecules [56]. Further investigation is needed to determine the exact mechanism of RNA uptake into CD4^+^ T cells.

Some caveats limit the interpretation of our findings: first only a few HIV-1 infected patients displayed high GPR15 expression on T cells and this did not correlate with viral load in the blood. An explanation for the observed GPR15 increase in a subset of HIV-1 infected patients could be potential co-infection with another virus which may lead to additional TLR3 triggering followed by GPR15 increase.

TLR expression on lymphocytes remains controversial in the literature but it is proposed that CD8^+^, similarly to CD4^+^ T cells, express TLRs 1–5 and 8–10 [30], [57]. Peripheral blood B cells express TLR 1, 2, 4, 6, 9 and 10 [31]. Other studies propose additional TLR3 expression in a subset of peripheral blood B cells [58] and upper respiratory mucosa B cells [59]. In our study we show that TLR3 triggering can up-regulate GPR15 also on CD8^+^ T cells and CD19^+^ B cells leading us to the conclusion that TLR3 must be expressed on both cell populations in the peripheral blood. If GPR15 is up-regulated only in a subset of B cells remains to be investigated.

The gut is the major site of HIV-1 replication and the gut mucosal homing receptor α4β7-integrin binds HIV-1 Env with high affinity and might be important for productive infection of CD4^+^ T cells [33], [34], [38], [39]. In light of these findings, it is noteworthy that we observed a high expression of GPR15 on intestinal CD4^+^ T cells especially those of the LP and a more prominent up-regulation of GPR15 expression on gut-homing CD4^+^ T cells as compared to CD4^+^ T cells homing to the lymph nodes. In line with our findings a recent study suggested a role of GPR15 for the homing of T cells particularly FoxP3^+^ Tregs to the colon laminar propria of mice [24]. In view of our consistent findings *in vitro* it is puzzling that we could not detect a substantial difference in GPR15 expression when comparing gut tissue from HIV-1 patients to uninfected controls. One possible explanation for this finding is that in the gut compartment many pathogens and concurrent inflammation or other signals which imprint for gut homing may contribute to the high expression of GPR15 on immune cells [24] thus overriding the effect of TLR3 stimulation by HIV-1 on up-regulation of GPR15. Alternatively, an increased turnover of GPR15 positive cells may take place in the gut of HIV-1 infected individuals through virus-induced apoptosis.

The second possibility is likely since GPR15 is postulated to have a role in apoptosis induction in the gut. Gp120-GPR15 interaction has been reported to be a possible cause for HIV-1 induced enteropathy [60] and peak levels of apoptosis in SIV-induced enteropathy have been associated with gp120 shedding in the gut [61]. The physiological role of GPR15 is difficult to investigate since its natural ligand has not been identified yet. However a recent study suggests a role of GPR15 induced Treg gut-homing in dampening the immune response and thereby preventing pathological inflammation using a GPR15 knock-out mouse model [24]. Our results show for the first time that innate immune activation through TLR3 stimulation up-regulates GPR15 expression *in vitro*. Up-regulation was most prominent on gut homing CD4^+^ T cells and the receptor was highly expressed on intestinal CD4^+^ T cells. These findings, together with the documented role of GPR15 in apoptosis and gut homing of T cells, might argue for an important role of this protein in inflammatory processes in the gut.
